# Applying intraoral scanner to residual ridge in edentulous regions: in vitro evaluation of inter-operator validity to confirm trueness

**DOI:** 10.1186/s12903-019-0918-y

**Published:** 2019-12-02

**Authors:** Akinori Tasaka, Yuuki Uekubo, Tomoharu Mitsui, Takao Kasahara, Takuya Takanashi, Shinya Homma, Satoru Matsunaga, Shinichi Abe, Masao Yoshinari, Yasutomo Yajima, Kaoru Sakurai, Shuichiro Yamashita

**Affiliations:** 1 Department of Removable Partial Prosthodontics, Tokyo Dental College, 2-9-18 Kandamisakicho Chiyoda-ku, Tokyo, 101-0061 Japan; 2 Oral Health Science Center, Tokyo Dental College, Tokyo, Japan; 3 Department of Prosthodontics, Matsumoto Dental University, Shiojiri, Japan; 4 Department of Oral and Maxillofacial Implantology, Tokyo Dental College, Tokyo, Japan; 5 Department of Anatomy, Tokyo Dental College, Tokyo, Japan; 6 Department of Removable Prosthodontics and Gerodontology, Tokyo Dental College, Tokyo, Japan

**Keywords:** Intraoral scanner, Optical impression, Edentulous, Free end saddles, Residual ridge

## Abstract

**Background:**

The purpose of this study was to investigate the trueness of intraoral scanning of residual ridge in edentulous regions during in vitro evaluation of inter-operator validity.

**Methods:**

Both edentulous maxillary and partially edentulous mandibular models were selected as a simulation model. As reference data, scanning of two models was performed using a dental laboratory scanner (D900, 3Shape A/S). Five dentists used an intraoral scanner (TRIOS 2, 3Shape A/S) five times to capture intraoral scanner data, and the “zig-zag” scanning technique was used. They did not have experience with using intraoral scanners in clinical treatment. The intraoral scanner data was overlapped with the reference data (Dental System, 3Shape A/S). Regarding differences that occurred between the reference and intraoral scanner data, the vertical maximum distance of the difference and the integral value obtained by integrating the total distance were analyzed.

**Results:**

In terms of the maximum distances of the difference on the maxillary model, the means of five operators were as follows: premolar region, 0.30 mm; molar region, 0.18 mm; and midline region, 0.18 mm. The integral values were as follows: premolar region, 4.17 mm^2^; molar region, 6.82 mm^2^; and midline region, 4.70 mm^2^. Significant inter-operator differences were observed with regard to the integral values of the distance in the premolar and midline regions and with regard to the maximum distance in the premolar region, respectively. The maximum distances of the difference in the free end saddles on mandibular model were as follows: right side, 0.05 mm; and left side, 0.08 mm. The areas were as follows: right side, 0.78 mm^2^; and left side, 1.60 mm^2^. No significant inter-operator differences were observed in either region.

**Conclusions:**

The present study demonstrated satisfactory trueness of intraoral scanning of the residual ridge in edentulous regions during in vitro evaluation of inter-operator validity.

## Background

The recent spread of digital dentistry has seen remarkable innovation in the capture of optical impressions using intraoral scanners, with three-dimensional (3D) full-color image scanning now possible. Development of a workflow to fabricate crown restorations using the acquired imaging data is already underway [1–3].

Various systems of computer-aided design (CAD)/computer-aided manufacturing (CAM) fabrication of complete dentures have been devised [4]. CAD/CAM systems have already been applied to denture base milling and artificial tooth attachment; denture base additive manufacturing and artificial tooth attachment; and milling of discs consisting of denture base and artificial tooth. Although limited to case reports of CAD/CAM fabrication of partial dentures, satisfactory results have been published for CAD/CAM framework fabrication using intraoral scanners to capture optical impressions [5–8]. The 3D data of the oral cavity is used to create the CAD data of the framework for a digital wax-up. Additive manufacturing is then performed based on the framework data to create the resin pattern, after which the pattern is invested and cast [9], and the framework is molded. Another method is to mold the framework using selective laser melting [10].

However, the approaches used in all of these systems are not equivalent to creating functional impressions using conventional impression materials. The difficulty involved in capturing optical impressions of viscoelastic bodies such as mucosa required for removable dentures has delayed the spread of intraoral scanner use in this field of dentistry. It should be noted that it is difficult to obtain data regarding the amount of tissue displacement of the residual mucous membrane and the functional morphology of mobile tissues such as the oral vestibular, lips, tongue and cheeks with an intraoral scanner.

The fabrication of removable dentures using an intraoral scanner has many advantages, such as reducing patient discomfort of impression taking, eliminating rubber allergies, the distortion of impression material and storing the scan data [11]. Although many studies have verified the trueness and precision of optical impressions captured using intraoral scanners for remaining teeth [12–15], many points remain to be clarified regarding precision for the residual ridge in edentulous regions [16–19]. In order to establish a workflow for CAD/CAM fabrication of removable dentures based on data acquired from intraoral scanners, the trueness and precision of intraoral scanning for residual ridge must be confirmed. The present study investigated the trueness of intraoral scanning regarding the residual ridge in edentulous regions for in vitro evaluation of inter-operator validity.

## Methods

### Simulation models

An edentulous maxillary model (G10FE-402 K, Nissin Dental Products Inc., Tokyo, Japan) and a partially edentulous mandibular model with free end saddles (P25-TP49, Nissin Dental Products Inc., Tokyo, Japan) were used as simulation models. The artificial mucosa made from silicone was attached on each simulation model. The free end saddles on mandibular model were Kennedy class I with missing bilateral molars and left second premolar. Rest seats were prepared on the distal proximal surface of the mandibular right second and left first premolars.

### Data acquisition and superimposition

A dental laboratory scanner (D900, 3Shape A/S, Copenhagen, Denmark) was used for 3D scanning of the maxillary and mandibular simulation models to acquire reference data. The D900 employs 5.0 MP cameras, and the scanner’s optical system has been optimized for speckle-free capture. Four cameras and new blue light-emitting diode technology had highly accurate color scanning at ±7 μm.

The simulation models were fitted to the SIMPLE MANIKIN III (Nissin Dental Products Inc., Tokyo, Japan) and attached to a dental chair with the Head Rest Mount SPMIII (Nissin Dental Products Inc., Tokyo, Japan). Five dentists each used an intraoral scanner (TRIOS 2, 3Shape A/S, Copenhagen, Denmark, https://www.3shape.com/en/support-docs) five times to capture optical impression data. They had not used intraoral scanners in clinical treatment. The “zig-zag” scanning technique was used in this study. After scanning, unnecessary information (islands and peninsulas) was trimmed and removed using the tool function.

**Fig. 1:**
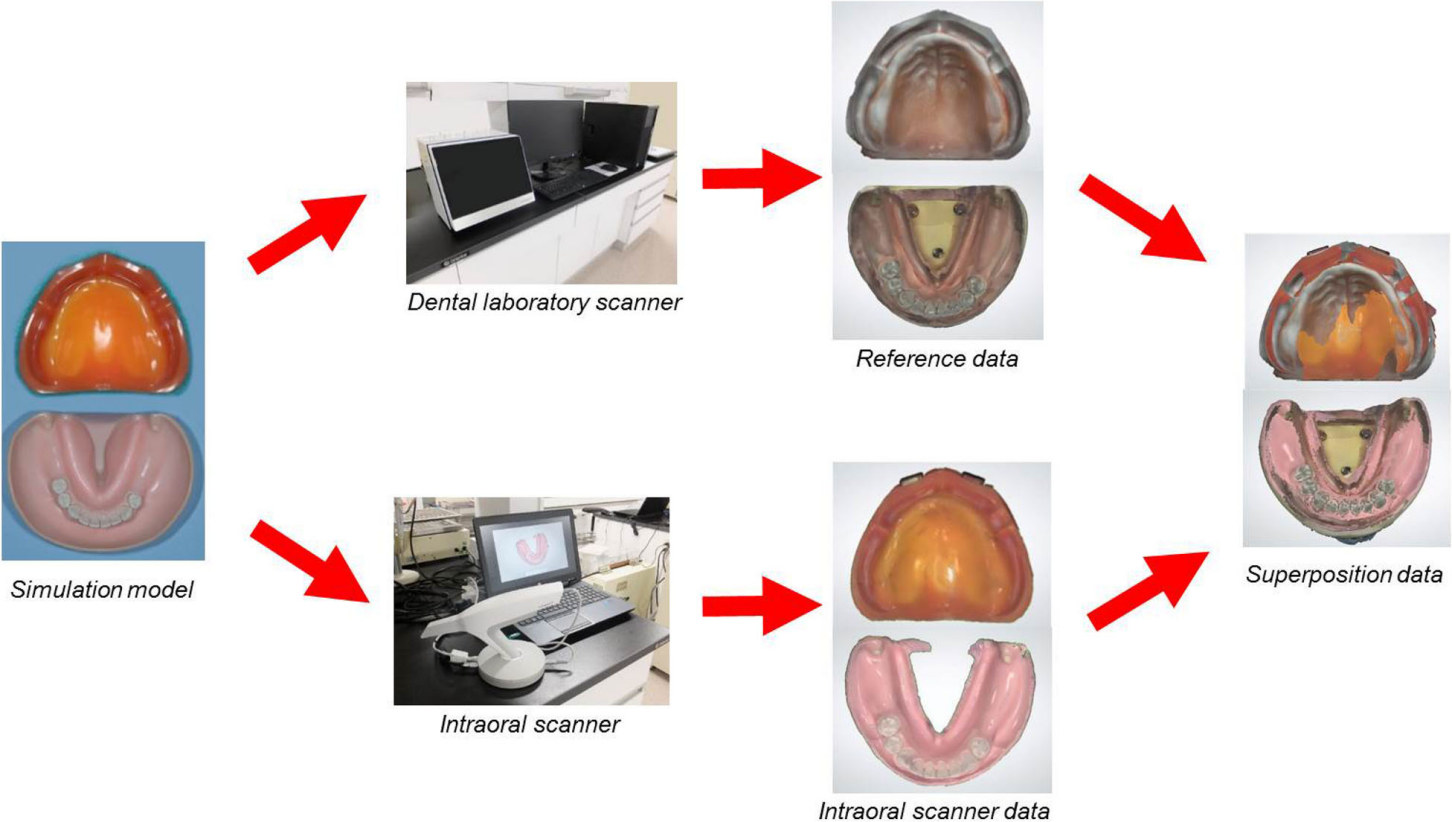
Flowchart of data capture and superposition. Dental laboratory scanner (D900; 3Shape). Intraoral scanner (Trios2; 3Shape)

The captured intraoral scanner data were imported into CAD software (Dental System, 3Shape A/S, Copenhagen, Denmark). Using the double scan technique of CAD software, intraoral scanner data were superimposed onto the reference data on the basis of the incisive papilla (1 point) and the top of the bilateral maxillary tubercles (2 points) for the edentulous maxillary model and of the incisal point (1 point) and the centers of the bilateral retromolar pads (2 points) for the free end missing mandibular model (Fig. 1).

### Trueness verification

**Fig. 2:**
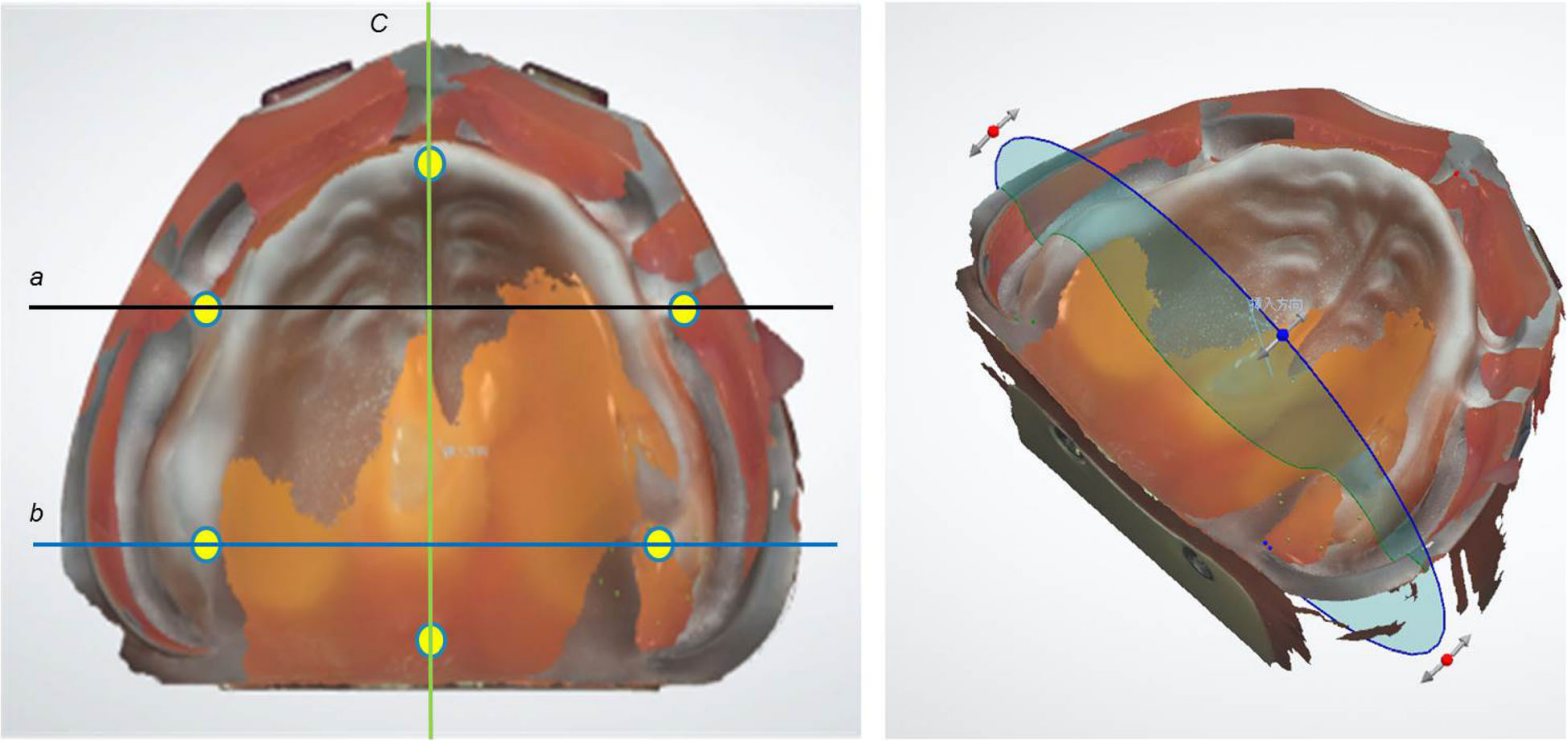
Defined site of analysis in edentulous maxilla and example of measurement in molar region. Left: Premolar region **a**: coronal section spanning bilateral buccal frenulum. Molar region (**b**): coronal section spanning points of bilateral maxillary tubercles. Midline region (**c**): sagittal section extending from center of incisive papilla to center of palatine foveola. Right: example of measurement in edentulous maxilla on computer display. (Molar region (**b**))

**Fig. 3:**
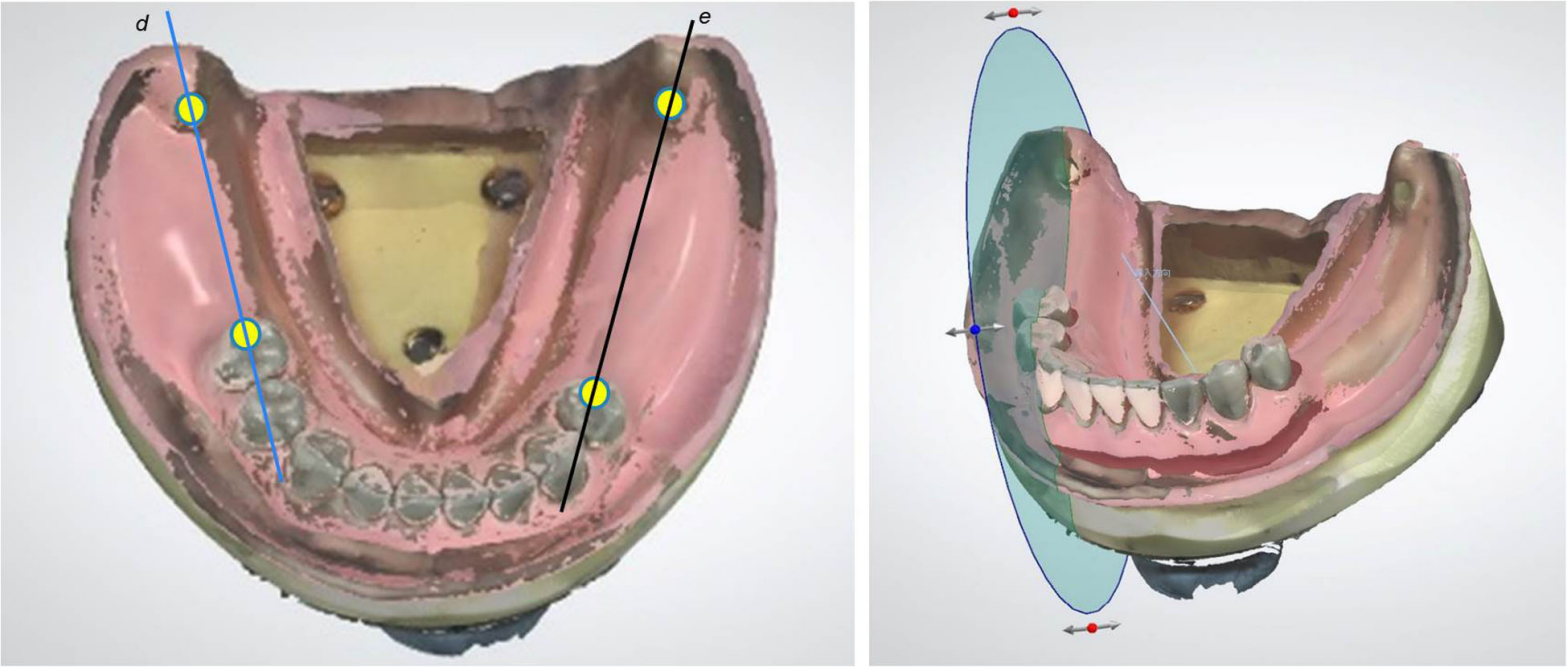
Defined site of analysis in partially edentulous mandible and example of measurement in molar region. Left; Right side (**d**): sagittal section extending from rest seat on right second premolar to center of retromolar pad. Left side (**e**): sagittal section extending from rest seat on left first premolar to center of retromolar pad. Right: example of measurement in partially edentulous mandible on computer display. (Right side (**d**))

Scanning data were acquired in several regions of each model in order to verify trueness. In the maxillary, these verification regions comprised a coronal section spanning the bilateral buccal frenulum (premolar region), a coronal section spanning the points of the bilateral maxillary tubercles (molar region), and a sagittal section extending from the center of the incisive papilla to the center of the palatine foveola (midline region) (Fig. 2). In the mandibular, these regions comprised a sagittal section extending from the rest seat on the right second premolar to the center of the retromolar pad (right side) and a sagittal section extending from the rest seat on the left first premolar to the center of the retromolar pad (left side) (Fig. 3).

**Fig. 4:**
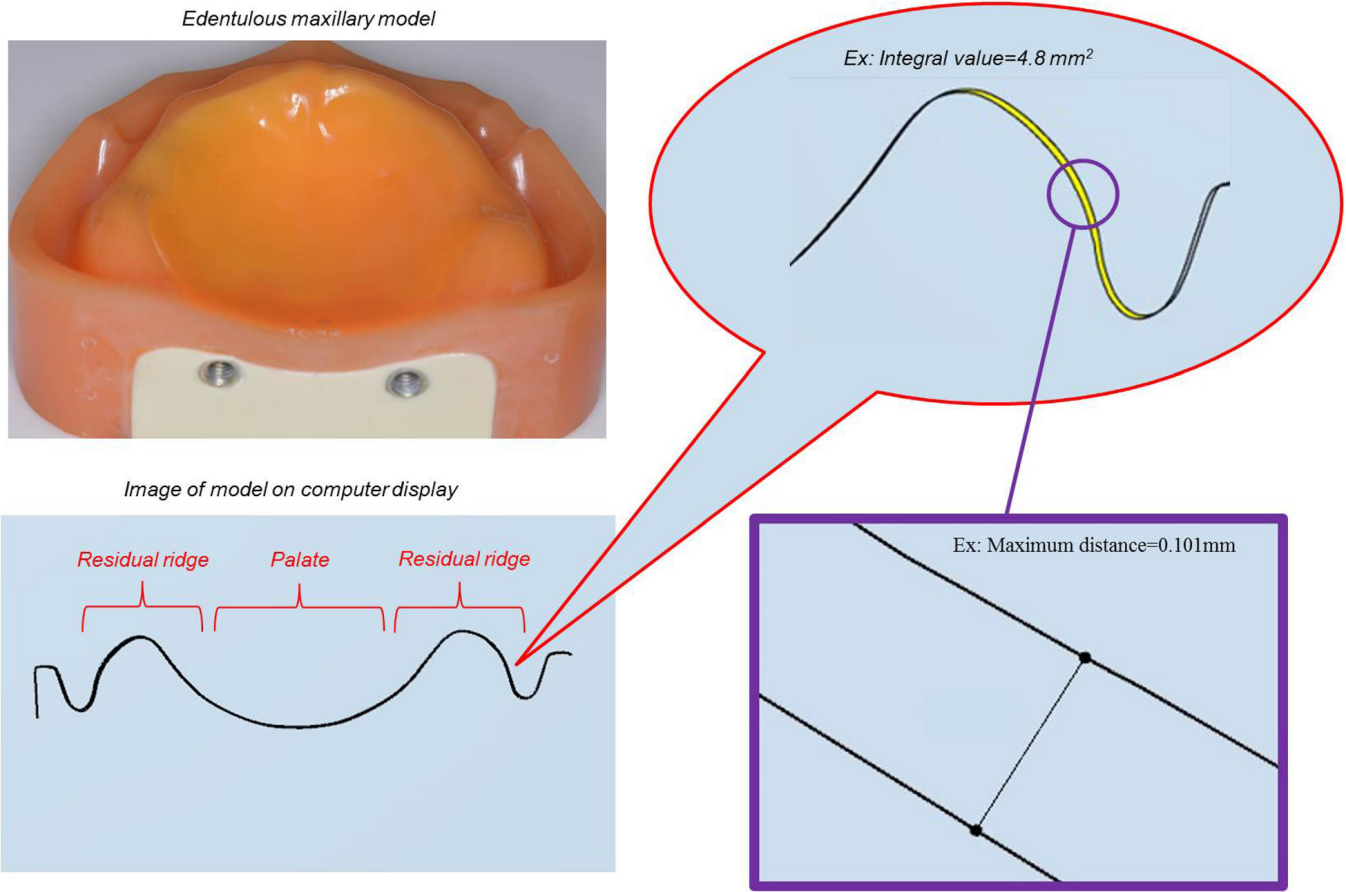
Summary of analysis items in edentulous maxilla. Left: Edentulous maxilla model and computer display of coronal section spanning points of bilateral maxillary tubercles. Right: Maximum distance (point to point) and integral value (surrounded by yellow) obtained by integrating total distance were analyzed using software (Dental system; 3shape)

**Fig. 5:**
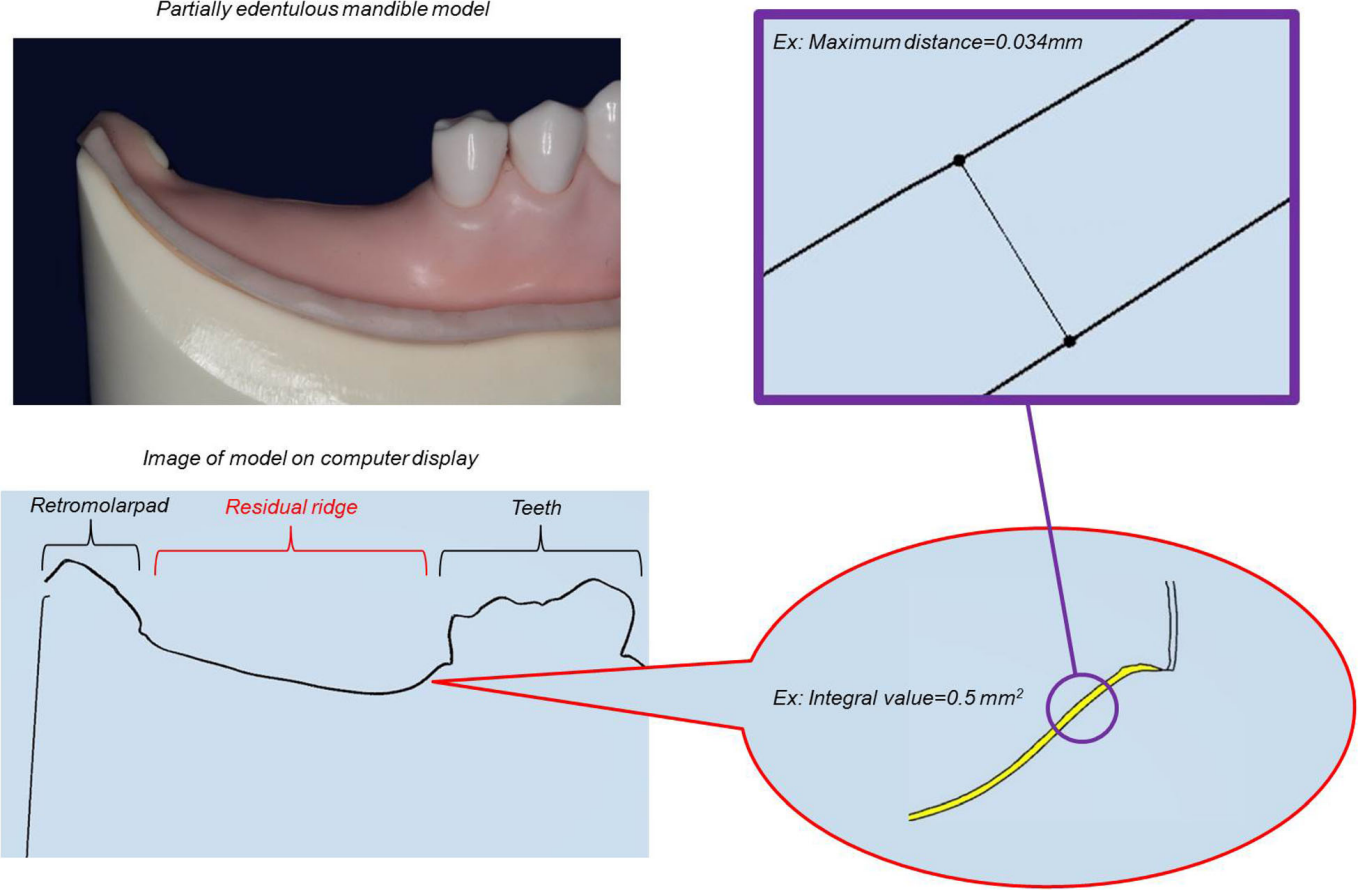
Summary of analysis items in partially edentulous mandible. Left: Partially edentulous model and computer display of sagittal section extending from rest seat on right second premolar to center of retromolar pad. Right: Maximum distance (point to point) and integral value (surrounded by yellow) obtained by integrating total distance were analyzed using software (Dental system; 3shape)

The amount of error between the reference data and intraoral scanner data in each verification region was measured, and the vertical maximum distance of the difference and the value obtained by integrating the total distance were analyzed using a two-dimensional cross section tool of the CAD software mentioned above (Figs. 4 and 5).

### Statistical analysis

The vertical maximum distance of the difference and the integral value in each verification region were analyzed using the Kruskal-Wallis test, while evaluation of inter-operator validity to confirm trueness was performed with the Steel-Dwass test for multiple comparisons. Statistical analyses were performed using SPSS version 22 (IBM, New York, NY) with significance set at *p* < 0.05.

## Results

**Fig. 6:**
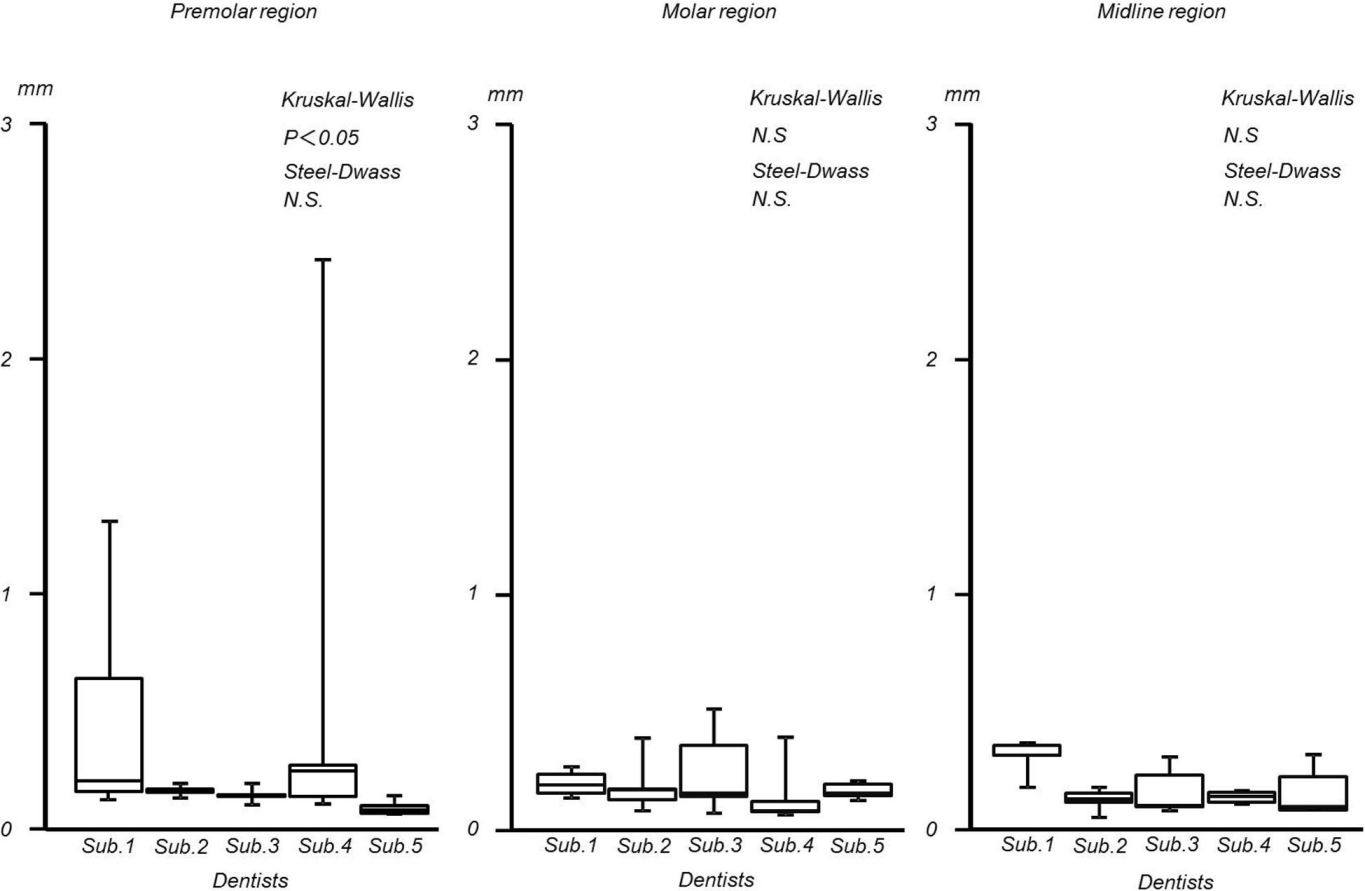
Maximum distance of edentulous maxilla

**Fig. 7:**
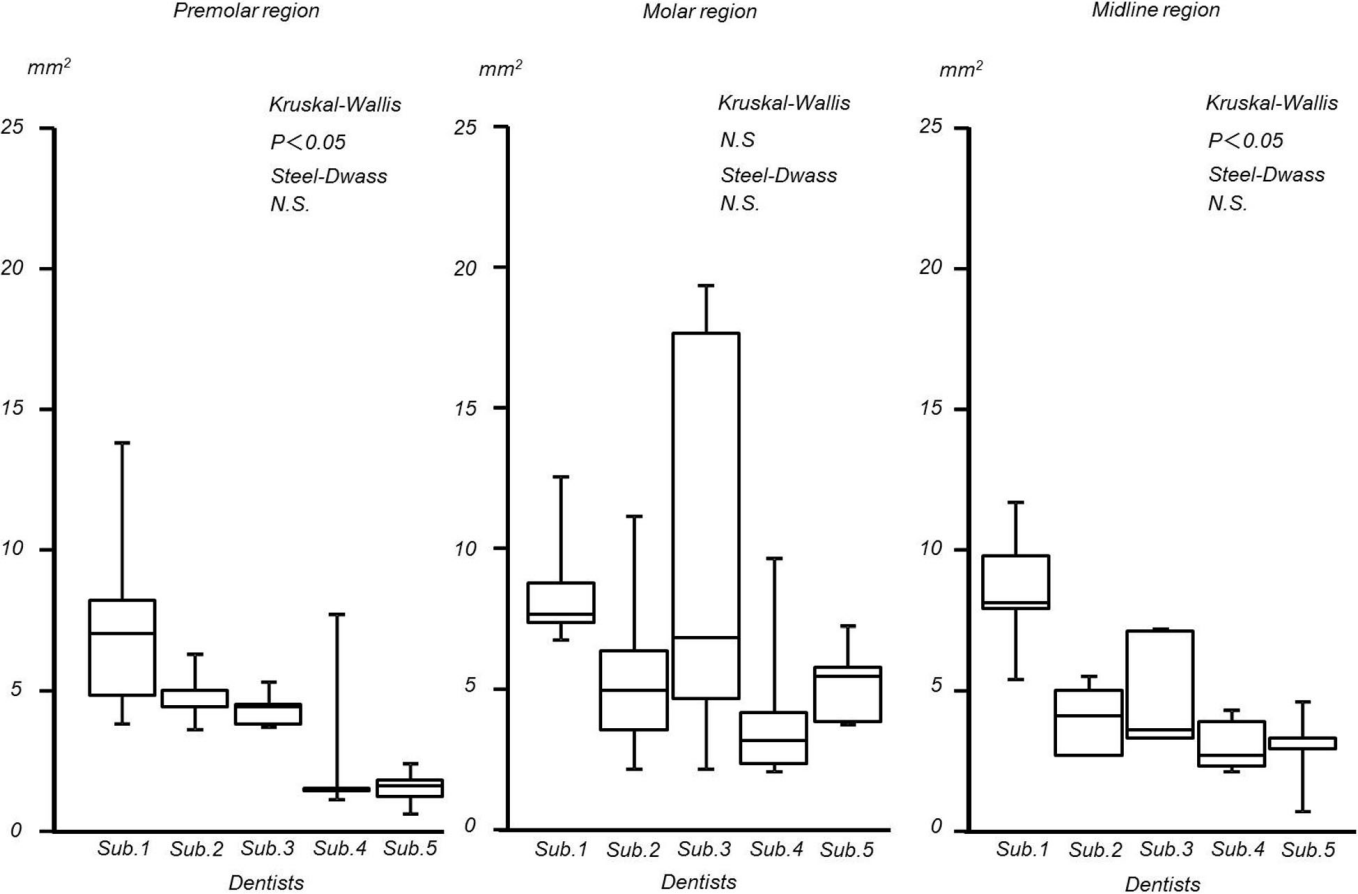
Integral value of edentulous maxilla

In the edentulous maxillary model, the maximum distances of the difference were as follows: premolar region; 0.30 ± 0.24 (mean ± standard deviation) mm, molar region; 0.18 ± 0.04 mm, and midline region; 0.18 ± 0.07 mm. (Interquartile range: premolar region; 0.01 to 0.481 mm, molar region; 0.04 to 0.08 mm, midline region; 0.04 to 0.14 mm). The integral values were as follows: premolar region; 4.17 ± 2.30 mm^2^, molar region; 6.82 ± 2.48 mm^2^, and midline region; 4.70 ± 2.30 mm^2^ (interquartile range: premolar region; 0.1 to 3.4 mm^2^, molar region; 1.4 to 13 mm^2^, midline region; 0.4 to 3.8 mm^2^). Significant inter-operator differences were observed in the premolar and midline regions with regard to the integral values and in the premolar region with regard to the maximum distances of the difference (Figs. 6 and 7).

**Fig. 8:**
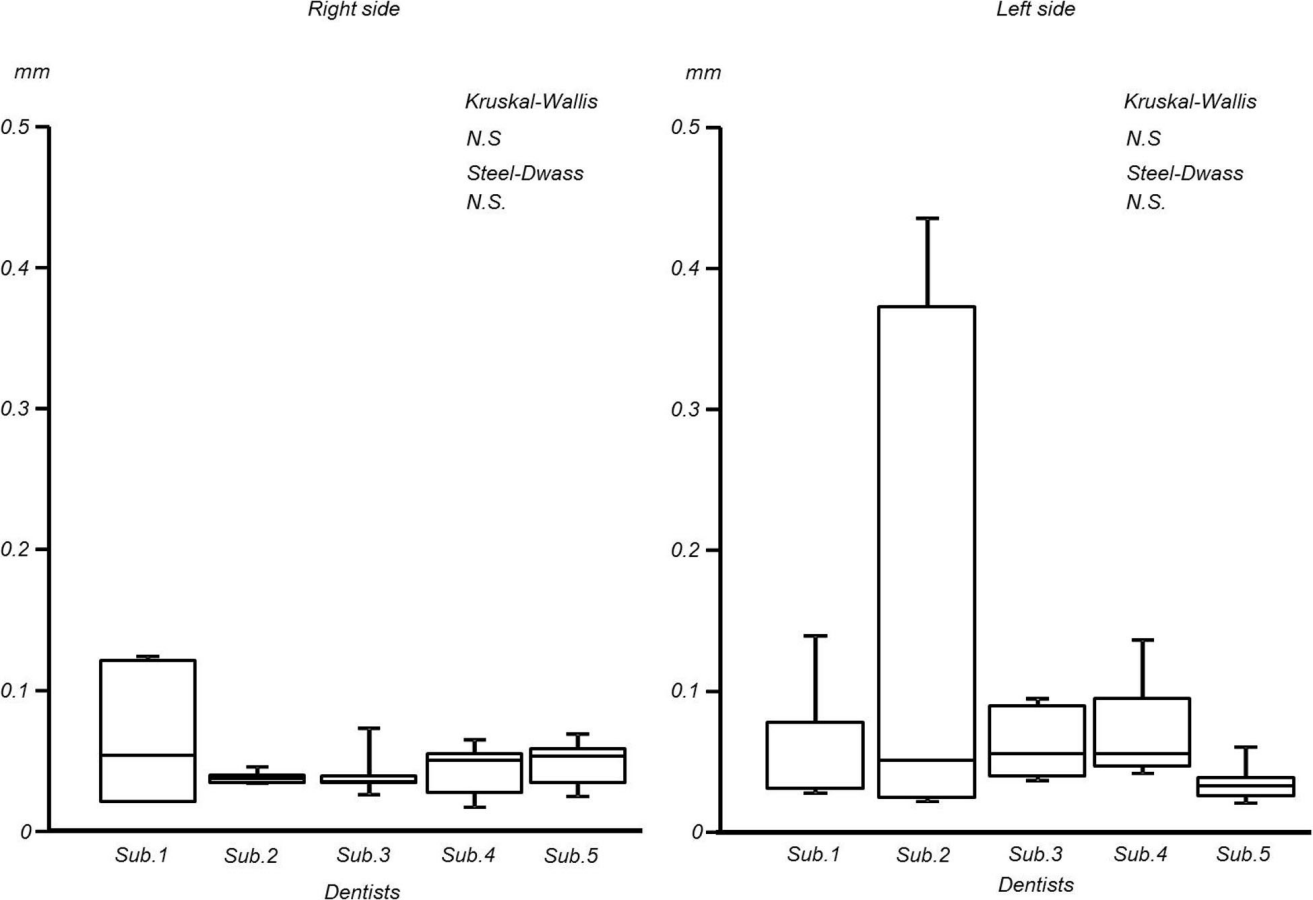
Maximum distance of mandibular free-end saddles

**Fig. 9:**
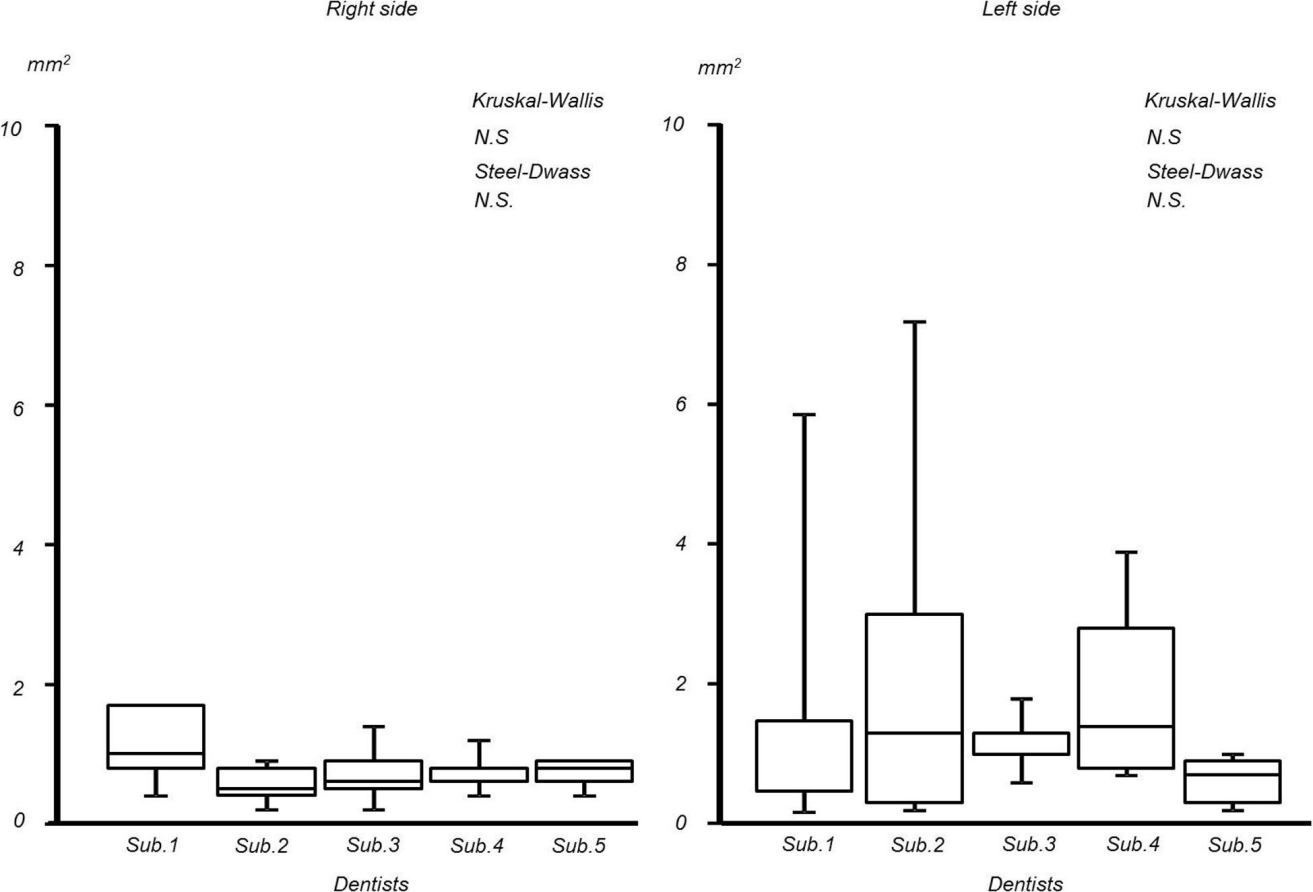
Integral value of mandibular free-end saddles

In the partially edentulous mandibular model, the maximum distances of the difference in the free end saddles on mandibular model were as follows: right side; 0.05 ± 0.01 mm and left side; 0.08 ± 0.05 mm (interquartile range: right side; 0.00 to 0.10 mm, left side; 0.01 to 0.35 mm). The integral values were as follows: right side; 0.78 ± 0.21 mm^2^ and left side; 1.60 ± 0.71 mm^2^ (interquartile range: right side; 0.2 to 0.9 mm^2^, left side; 0.3. to 2.7 mm^2^). No significant inter-operator differences were observed for the maximum distances of the difference or the integral value s in either region (Figs. 8 and 9).

## Discussion

Many studies have verified the trueness and precision of intraoral scanners that use methods such as a confocal method with a light-emitting diode light source or active wavefront sampling. The intraoral scanner used in the present study applies the former system and comparatively good trueness and precision has been consistently demonstrated in previous studies [20–24].

In the present study, the maxillary and mandibular simulation models were based on an edentulous jaw and partial edentulous arch, respectively. Reference data were acquired from a dental laboratory scanner, which has greater accuracy (±7 μm) than an intraoral scanner. Park et al. reported that the root mean square value of the laboratory scanner (47.5 ± 1.6 μm) is smaller than the intraoral scanner (343.4 ± 56.4 μm) in fully dentulous individuals [25]. This high accuracy is possible because the measurement target is fixed and natural light is blocked, enabling data to be acquired from a variety of angles with a high-performance camera [23, 26].

The regions selected for verification of precision of intraoral scanning centered on the support area in the maxillary model and on the standard area for placement of artificial tooth arrangements in the mandibular model. The method of superimposition in this study was feature based. Using the double scan technique of CAD software, the data were aligned by 3 points. The choice of points was for the operator to decide. Three points of the characteristic anatomical structures of the edentulous maxillary and the free end missing mandibular model were selected. Significant inter-operator differences in errors in the intraoral scanning were observed in the premolar region (maximum distances of the difference and integral values) and midline region (integral values) in the edentulous maxillary model. Poorly traceable structures and a flat shape are characteristic on the palatal, suggesting that it would be difficult to stitch the image [16]. Although the edentulous maxillary model used in the present study was equivalent to the American College of Prosthodontists Type A jaw [27], factors such as residual ridge morphology, palatal depth, and the presence or absence of palatal tori may have affected the results [28, 29]. Conversely, no significant inter-operator differences were observed in errors in the intraoral scanning of either the left or right side of the free end saddles on mandibular model. This suggests that the operator effect on optical impressions in the free end saddles on mandible is small. However, the interquartile range for the maximum values of the difference and the integral values tended to be larger on the left side than on the right side of the free end saddles on mandibular model. This is likely because the range in the free end saddles on mandibular model was mesiodistally longer on the left than the right side. Compared to teeth, the residual ridge has fewer anatomical elements, which may have affected image-stitching errors [16]. Similar results were obtained in an in vitro study of repeatability of intraoral scanner for the partially edentulous [30]. Kim et al. reported that trueness and precision of intraoral scanner were improved using an artificial landmark in the long edentulous region [31]. The intraoral scanner is affected by various conditions, and Albdour et al. suggest that different light reflections on teeth and mucous membranes affect accuracy [32]. Therefore, no significant inter-operator differences were observed, and as the maximum distances (0.04 to 0.60 mm) of the difference between the intraoral scanner data and reference data were the same or lower than the amount of tissue displacement (0.70 to 1.00 mm from results of in vivo study) [33], with practice, operators can bring these errors to within a clinically acceptable range. No significant differences were observed between conventional impression and intraoral scanner, and there were no clinically significant effects on fabrication of removable denture [11, 34, 35]. This suggests that intraoral scanning of edentulous areas could achieve satisfactory capture by the operator.

There are two major impediments to the clinical application of optical impressions of residual mucous membrane. First, as a viscoelastic body, the residual mucous membrane is susceptible to tissue displacement. The impressions acquired of the residual mucous membrane in the present study were anatomic impressions. However, relief and pressure are possible after data digitalization. Okubo et al. reported the CAD/CAM fabrication of a mandibular complete denture in which digital relief of the mental foramen was performed [36]. In the partial edentulous arch, in order to compensate for the difference in the amount of tissue displacement between the residual teeth and the residual mucous membrane, digital pressurization of the acquired residual mucous membrane data is required. However, a simple method of acquiring data regarding the amount of tissue displacement of the residual mucous membrane has yet to be established [37]. Second, it is difficult to acquire data regarding the functional morphology of mobile tissues such as the oral vestibule, lips, tongue, and cheeks with an intraoral scanner [38]. The development of methods of border molding using intraoral scanners and devices that can acquire data in mobile regions are awaited. Due to these issues, at present, intraoral scanners are used in edentulous patients for preliminary impressions, after which individual trays are fabricated based on those data and functional impressions are made using conventional methods [39]. In free end saddles, intraoral scanners can be used to make anatomic impressions of residual teeth and residual mucous membrane, from which data a metal framework is fabricated and functional impressions are made using the altered cast technique [40].

A limitation of this study was that the model we selected had a completely different behavior than human soft tissues. Moreover, only one type of scanner was used and the operators captured only five data sets. Imburgia et al. reported that the type of scanner affected the scanning accuracy of the missing tooth pattern [41]. For verification of trueness of intraoral scanning on specific region and limited tooth missing patterns, further study is required to investigate the use of other scanners, methods and conditions.

## Conclusions

The present study demonstrated satisfactory trueness of intraoral scanning of residual ridge in edentulous regions during in vitro evaluation of inter-operator validity. The difference between the intraoral scanner data and reference data were the same or lower than the amount of tissue displacement. However, it was revealed that the lack of traceable structures and smooth surfaces, such as the palatal region, and/or long free end saddles, affected the trueness. If care is taken regarding these issues, the present study shows that optical impressions can be applied to the residual ridge of edentulous regions.

## Data Availability

Data are available from the corresponding author after approval by all authors.

## Abbreviations

3D: Three-dimensional
CAD/CAM: Computer-aided design / computer-aided manufacturing
